# ZO-2 determines cell membrane localization of receptor NTCP and supports hepatitis B virus infection

**DOI:** 10.1128/mbio.02704-25

**Published:** 2026-03-23

**Authors:** Hironori Nishitsuji, Saori Yoshimura, Daijiro Konno, Taro Tachibana, Masaya Sugiyama, Manabu Yamasaki, Masashi Mizokami, Takayuki Murata, Kunitada Shimotohno

**Affiliations:** 1 Department of Virology, Fujita Health University School of Medicine 89305, Toyoake, Japan; 2 Center for Infectious Disease Research, Research Promotion Headquarters, Fujita Health University 12695, Toyoake, Japan; 3 Cell Engineering Corporation, Osaka, Japan; 4 Department of Energy and Materials, Faculty of Science and Engineering, Kindai University 12872, Osaka, Japan; 5 Department of Bioengineering, Graduate School of Engineering, Osaka Metropolitan University 12936, Osaka, Japan; 6 Department of Viral Pathogenesis and Controls, National Institute of Global Health and Medicine, Japan Institute for Health Security, Ichikawa, Japan; 7 Institute of Microbial Chemistry (BIKAKEN), Shinagawa-ku, Tokyo, Japan; 8 Molecular and Cellular Glycoproteomics Research Group, Cellular and Molecular Biotechnology Research Institute, National Institute of Advanced Industrial Science and Technology 666244 , Tsukuba, Japan; Princeton University, Princeton, New Jersey, USA

**Keywords:** HBV, NTCP, ZO-2

## Abstract

Hepatitis B virus (HBV) infects hepatocytes by using sodium taurocholate cotransporting polypeptide (NTCP) as a receptor. NTCP can render non-permissive hepatocyte cell lines such as HepG2 permissive to HBV infection. However, only a few reports investigate the NTCP function in HBV infection beyond virus binding. Here, we performed immunopurification followed by liquid chromatography-tandem mass spectrometry to identify ZO-2 (aka TJP2) as a novel NTCP-binding protein. Knockdown or knockout of ZO-2 in NTCP-transduced HepG2 cells decreased the amount of NTCP at the cell surface, leading to reductions in cellular attachment and infection by HBV. Incubation of cells with HBV surface molecules preS1 resulted in the dissociation of NTCP from ZO-2 and in the formation of NTCP-preS1-actin complexes that were internalized into the cell. Latrunculin A, an inhibitor of actin polymerization, suppressed the preS1 internalization into hepatocytes and HBV infection. In conclusion, ZO-2, together with actin, regulates the function of NTCP as a receptor of HBV, providing a new model for HBV-cell interactions and virus internalization.

**IMPORTANCE:**

Although a number of candidates have been reported to bind to the hepatitis B virus (HBV) envelope, accumulating evidence indicates that NTCP is accepted as a functional receptor for HBV infection. Thus, NTCP is an attractive target for antiviral therapies. Here, we showed that ZO-2 interacts with NTCP. The silencing of ZO-2 decreased HBV infection, whereas ZO-1 and ZO-3 knockdown had no effect on HBV infection. Moreover, knockout of ZO-2 induced the downregulation of NTCP from the cell surface. This aberrant NTCP localization causes the reduction of the half-life of NTCP in ZO-2 knockout cells. PreS1 treatment or HBV infection disrupted the NTCP/ZO-2 complex through the dissociation of the actin-binding domain of ZO-2, leading to internalization of a newly formed preS1/NTCP/actin complex into the cell. The actin polymerization inhibitor latrunculin A suppressed HBV infection. These results suggest that ZO-2 regulates cell surface localization of NTCP.

## INTRODUCTION

Hepatitis B virus (HBV) efficiently replicates within hepatocytes, promoting liver diseases such as cirrhosis and hepatocellular carcinoma (1). The membrane envelope of HBV contains large (L), middle (M), and small (S) surface proteins, which are encoded from a single gene and only differ in their N-termini. The S form comprises the S domain, while the L and M forms comprise the combined preS1+preS2+S domains and combined preS2+S domains, respectively. At the initial stage of infection, HBV attaches to cell surface heparan sulfate proteoglycans (HSPGs) by means of the antigenic loop in the S domain (2). Non-specific binding of HBV to cells may additionally be promoted by ApoE, a ligand for low-density lipoprotein receptors, and HSPGs that were found associated with the HBV envelope, as an ApoE-specific monoclonal antibody could block infection (3). While the binding of HBV to HSPGs is low affinity and not restricted to hepatocytes, the cell specificity of HBV infection involves the preS1 domain of its surface protein. The preS1 domain, constituting the N terminus of the L-protein, is myristoylated and important for HBV entry (4). The preS1 domain specifically binds to permissive cells such as HepaRG and human primary hepatocytes, but not to non-permissive cells such as Huh7 and HepG2 cells (5).

A receptor for the binding of preS1 to hepatocytes and HBV entry into cells is the hepatocyte-specific bile acid transporter sodium taurocholate cotransporting polypeptide (NTCP), and the expression of NTCP in non-permissive cell lines such as HepG2 or Huh7 cells confers susceptibility to HBV and hepatitis D virus (HDV) (6). HDV is an RNA virus that uses HBV envelope proteins for cell entry. HDV can also infect transgenic mice expressing human NTCP *in vivo* (7), and anti-NTCP antibody suppresses HDV infection of HepG2-NTCP cells and NTCP transgenic mice (8). The high-affinity binding of preS1 to NTCP (6) is regarded as an initial step for HBV entry (9). Viral particle-NTCP complexes are sorted to the endocytic pathways by clathrin-associated adaptor protein complexes (10, 11).

NTCP is a transmembrane transporter protein, and many proteins of this category have a propensity for oligomerization (12). The structure of NTCP involved in transporting bile salts is believed to be a homodimer, although each subunit retains transport capacity (13). NTCP also forms oligomers, possibly homodimers, at the cell surface after binding to preS1 or HBV, followed by collective internalization of NTCP with the preS1 or viral particles into the cell (14). Epidermal growth factor receptor (EGFR) can, by interaction with NTCP, participate as an important cofactor for HBV entry, resulting in enhanced cellular internalization of HBV together with NTCP and EGFR (15). However, the cellular mechanisms regulating NTCP expression and internalization dynamics during HBV infection remain to be elucidated. Despite the clinical importance of HBV, progress in this field has been limited due to a lack of understanding of the molecular mechanisms regulating viral entry into hepatocytes.

In the present study, ZO-2 (zonula occludens 2; aka tight junction protein 2, or TJP2) was identified as a novel interacting partner of NTCP and shown to support NTCP location at the cell membrane and HBV infection. HBV infection, or preS1 treatment, was found to induce two processes critical for cellular internalization of the virus, namely (i) the disruption of NTCP/ZO-2 interaction, followed by (ii) the binding of NTCP to F-actin. Thus, the function of NTCP as a receptor for HBV involves NTCP interactions with ZO-2 and actin.

## RESULTS

### Identification of ZO-2 as an NTCP-binding protein

**Fig 1:**
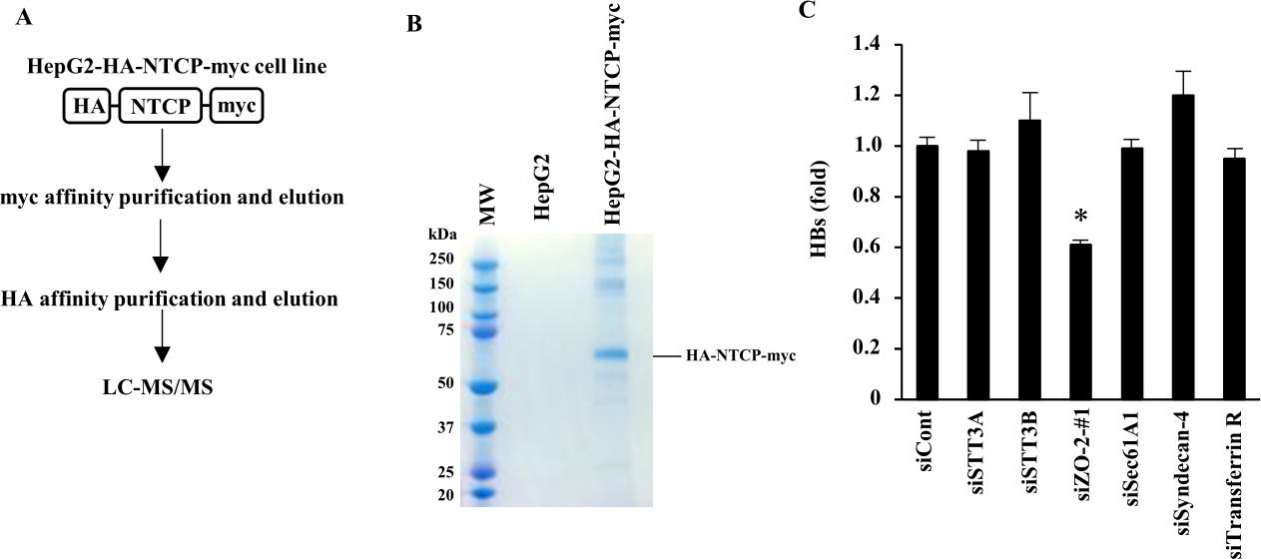
Identification of ZO-2 as a binding partner of NTCP. (**A**) Schematic of the experimental design for identifying NTCP-binding partners. HepG2 cells were transduced with a lentiviral vector encoding NTCP tagged with HA and myc epitopes at the N- and C-termini, respectively. The HA-NTCP-myc fusion protein was first purified using an anti-myc antibody and eluted with a myc peptide. The eluate was then subjected to a second purification step using anti-HA antibody-conjugated magnetic beads, followed by elution with an HA peptide. The resulting immunocomplex was analyzed by LC-MS/MS. (**B**) Eluate samples from panel A were subjected to SDS-PAGE and stained with Coomassie Brilliant Blue. (**C**) HepG2-NTCP cells were transfected with the indicated siRNA. At 2 days after transfection, cells were infected with HBV in the presence of 4% PEG8000 and 2% DMSO. The level of HBs was measured 9 days after infection. The data are presented as a mean ± s.d. (*n* = 3). ＊, *P* < 0.05.

To establish a system for identifying proteins that bind to NTCP, HepG2 cells were transduced to express HA- and myc-tagged NTCP (HA-NTCP-myc). Extracts were prepared from HepG2 and HepG2-HA-NTCP-myc cells. HA-NTCP-myc was purified by two consecutive immunopurifications, sequentially targeting the myc and HA tags (Fig. 1A). Purified NTCP-associated proteins were analyzed by SDS-polyacrylamide gel electrophoresis (PAGE) followed by Coomassie Brilliant Blue staining (Fig. 1B) and by liquid chromatography-tandem mass spectrometry (LC-MS/MS) (Table 1). Among the proteins identified by mass spectrometry (Table 1) were EGFR, already known as an NTCP-binding protein (15), and clathrin and AP-2, known to participate in clathrin-mediated endocytosis of HBV (10, 11). For further analysis, we selected STT3A, STT3B, ZO-2, Sec61A1, Syndecan-4, and Transferrin receptor, which had not been associated with a role in HBV infection previously. Their importance for HBV infection was estimated by the impact of their individual gene silencing through siRNA treatment of HepG2 cells transfected for myc-tagged NTCP (“HepG2-NTCP” cells) (Fig. S1). Among the investigated genes, only the silencing of ZO-2 significantly reduced HBV infection, as measured by the levels of HBV S antigen (HBs) in the culture supernatants 9 days after infection (Fig. 1C).

**TABLE 1:** Identification of cellular proteins as a binding partner of NTCP^*a*^

Gene name	Pep.	Cov. (%)
ZO-2	32	27
Transferrin R	14	7.6
STT3A	6	8.1
STT3B	5	6.4
Sec61B	2	27
Syndecan4	2	11
EGFR	2	1.7
CLTC	19	11
CLTA	6	17
CLTB	7	23
AP2A1	13	10
AP2B1	11	14
UB2G2	1	5
PSMD8	1	2
2AAB	1	2.6
Histone H1.0	1	5.2
60S ribosomal protein L29	1	9.4
Nup133	4	3
TRIM71	3	3.7
Ubiquitin carboxyl terminal hydrolase 10	2	1.9
UBR4	4	0.69

^*a*^ The number of identified peptides (Pep.) and percentage of total protein sequences covered (Cov. [%]) are shown.

### ZO-2 deficiency leads to intracellular localization and instability of NTCP

**Fig 2:**
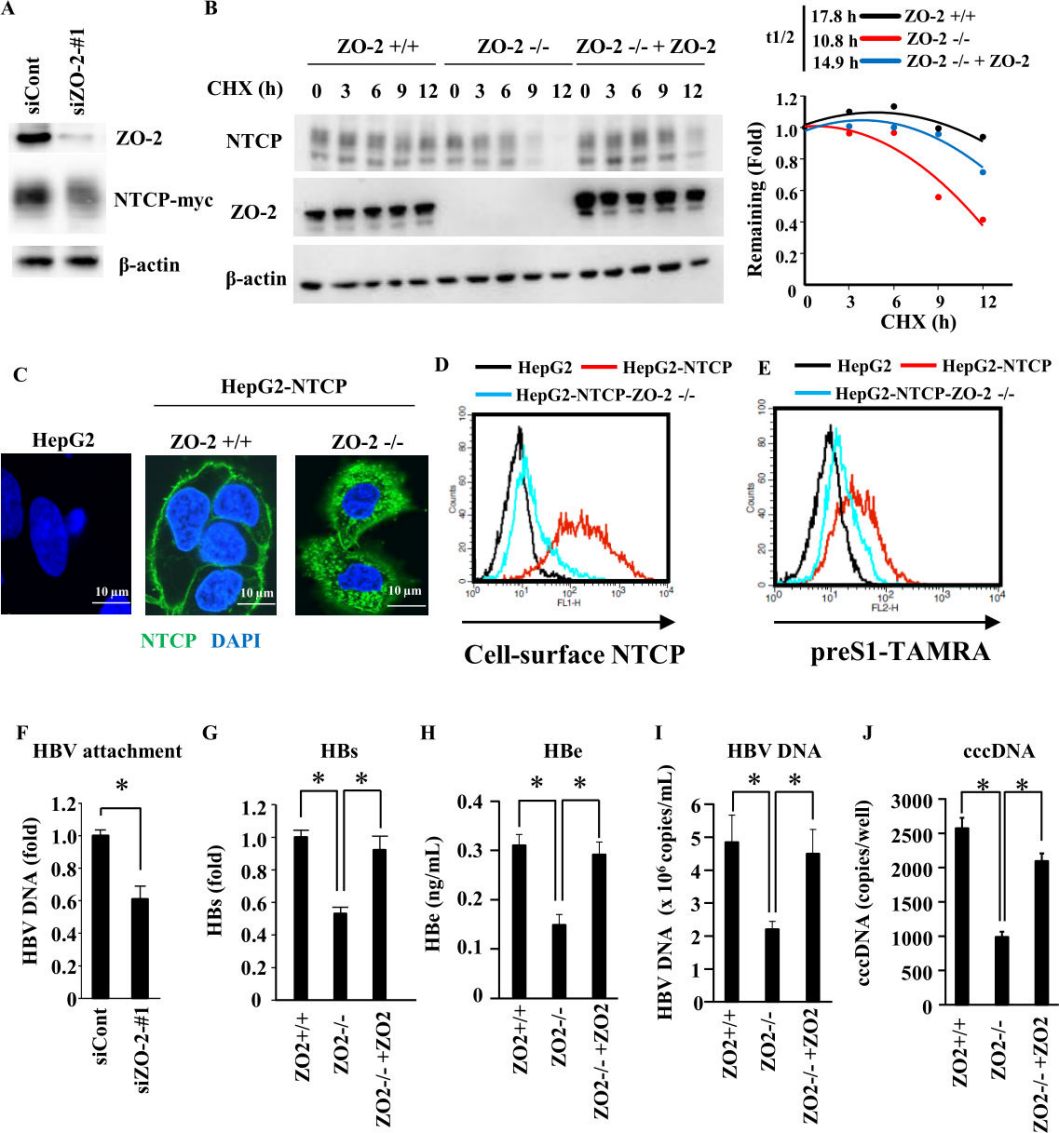
ZO-2 affects the stability of NTCP. (**A**) HepG2-NTCP cells were transfected with the indicated siRNA. At 48 h after transfection, cell lysates were analyzed by western blot using anti-NTCP antibody, anti-ZO-2 antibody, and anti-β-actin antibody. (**B**) ZO-2^+/+^ HepG2-NTCP, ZO-2^-/-^ HepG2-NTCP, and ZO-2^-/-^+ZO-2 HepG2-NTCP cells were treated with 100 μg/mL of cycloheximide for the indicated times. Cell lysates were analyzed by western blotting using anti-NTCP antibody, anti-ZO-2 antibody, and anti-β-actin antibody (left panel). The abundance of NTCP protein at various time points was compared to the level when cycloheximide treatment was initiated (*t* = 0 h, relative abundance of NTCP protein = 1.0) (right panel). (**C and D**) The localization of NTCP in ZO-2^+/+^ HepG2-NTCP cells and ZO-2^-/-^ HepG2-NTCP cells was compared by fluorescence microscopy after fluorescent staining of NTCP (green); cell nuclei were stained with 4′,6-diamidino-2-phenylindole (DAPI) (blue) (**C**). ZO-2^+/+^ HepG2-NTCP cells and ZO-2^-/-^ HepG2-NTCP cells were fluorescently stained for NTCP and analyzed by flow cytometer (**D**). (**E**) Cells were treated with 100 nM preS1-TAMRA. At 60 min after treatment, cells were analyzed by flow cytometer. (**F**) HepG2-NTCP cells were transfected with the indicated siRNA. At 48 h after transfection, cells were infected with HBV in the presence of 4% PEG8000 and 2% DMSO at 4°C for 3 h and then washed with PBS. The level of cell-surface HBV DNA was measured by real-time PCR. The data are presented as a mean ± s.d. (*n* = 5). (**G–J**) Parental HepG2-NTCP and ZO-2^-/-^ HepG2-NTCP cells transduced with lentivirus expressing either an empty vector or wild-type ZO-2 were infected with HBV in the presence of 4% PEG8000 and 2% DMSO. HBs (**G**), HBe (**H**), HBV DNA (**I**), and cccDNA (**J**) levels were measured 9 days after infection. The data are presented as a mean ± s.d. (*n* = 3). ＊, *P* < 0.05.

Next, we assessed the importance of ZO-2 for the localization and abundance of NTCP in hepatocytes. Knockdown of ZO-2 by siRNA treatment of HepG2-NTCP cells reduced the level of NTCP (Fig. 2A). To investigate whether ZO-2 regulates NTCP stability, we generated ZO-2 knockout HepG2-NTCP cells using CRISPR/Cas9 and performed a cycloheximide chase assay, in which cycloheximide was used to inhibit protein synthesis (Fig. 2B). The half-life of NTCP protein was significantly shorter in ZO-2^-/-^ HepG2-NTCP cells than in ZO-2^+/+^ HepG2-NTCP cells. The apparent stability of NTCP protein was mostly restored in ZO-2^-/-^ HepG2-NTCP cells after exogenous expression of ZO-2 to ZO-2^-/-^HepG2-NTCP cells (ZO-2^-/-^+ZO-2). Moreover, immunocytochemistry analysis comparing ZO-2^+/+^ HepG2-NTCP and ZO-2^-/-^ HepG2-NTCP revealed the importance of ZO-2 for NTCP localization, as NTCP was strongly associated with the cell membrane in the ZO-2^+/+^ cells, while predominantly located intracellularly in the ZO-2^-/-^ cells (Fig. 2C). Flow cytometry analysis confirmed this finding, as strong NTCP surface expression was only detected for ZO-2^+/+^ HepG2-NTCP cells (Fig. 2D).

These results suggest that ZO-2 stabilizes NTCP protein by delivering and/or retaining it at the cell surface. The fact that NTCP is a heavily glycosylated protein (16) and that, regardless of ZO-2 knockdown, NTCP has the same apparent size and thus must be similarly glycosylated and have undergone the same intracellular transport suggests that ZO-2 is involved in retaining NTCP at the cell surface rather than in its transport.

### ZO-2 depletion reduces attachment of preS1 peptide and HBV to the cell surface and HBV infection

To examine whether depletion of ZO-2 affects the binding of preS1 peptide to the cell surface, association of TAMRA-labeled myristoylated preS1 peptide (preS1-TAMRA) with HepG2-NTCP cell lines was analyzed by flow cytometry. A considerable reduction in the attachment of preS1-TAMRA to ZO-2^-/-^ HepG2-NTCP cells compared to ZO-2^+/+^ HepG2-NTCP cells was observed (Fig. 2E). In addition, the attachment of HBV particles to HepG2-NTCP cells was reduced after siRNA knockdown of ZO-2, as revealed after virus incubation at 4°C for 3 h, followed by washing and PCR quantification of the HBV DNA that remained associated with the cells (Fig. 2F). Finally, the effect of ZO-2 depletion on HBV production was measured by comparing HBV amounts in the cell supernatants. The ZO-2^-/-^ HepG2-NTCP cells exhibited reduced HBs (Fig. 2G), HBe (Fig. 2H), extracellular HBV DNA (Fig. 2I), and HBV cccDNA (Fig. 2J) compared to the parental HepG2-NTCP cells, but virus infection was rescued by exogenous expression of ZO-2. In summary, ZO-2 knockdown or knockout resulted in reduced attachment of the preS1 peptide (Fig. 2E), HBV attachment (Fig. 2F), and HBV infection (Fig. 2G through J), possibly all representing the same phenomenon caused by reduced levels of NTCP at the cell surface.

### ZO-2, but not ZO-1 or ZO-3, plays a role in HBV infection

**Fig 3:**
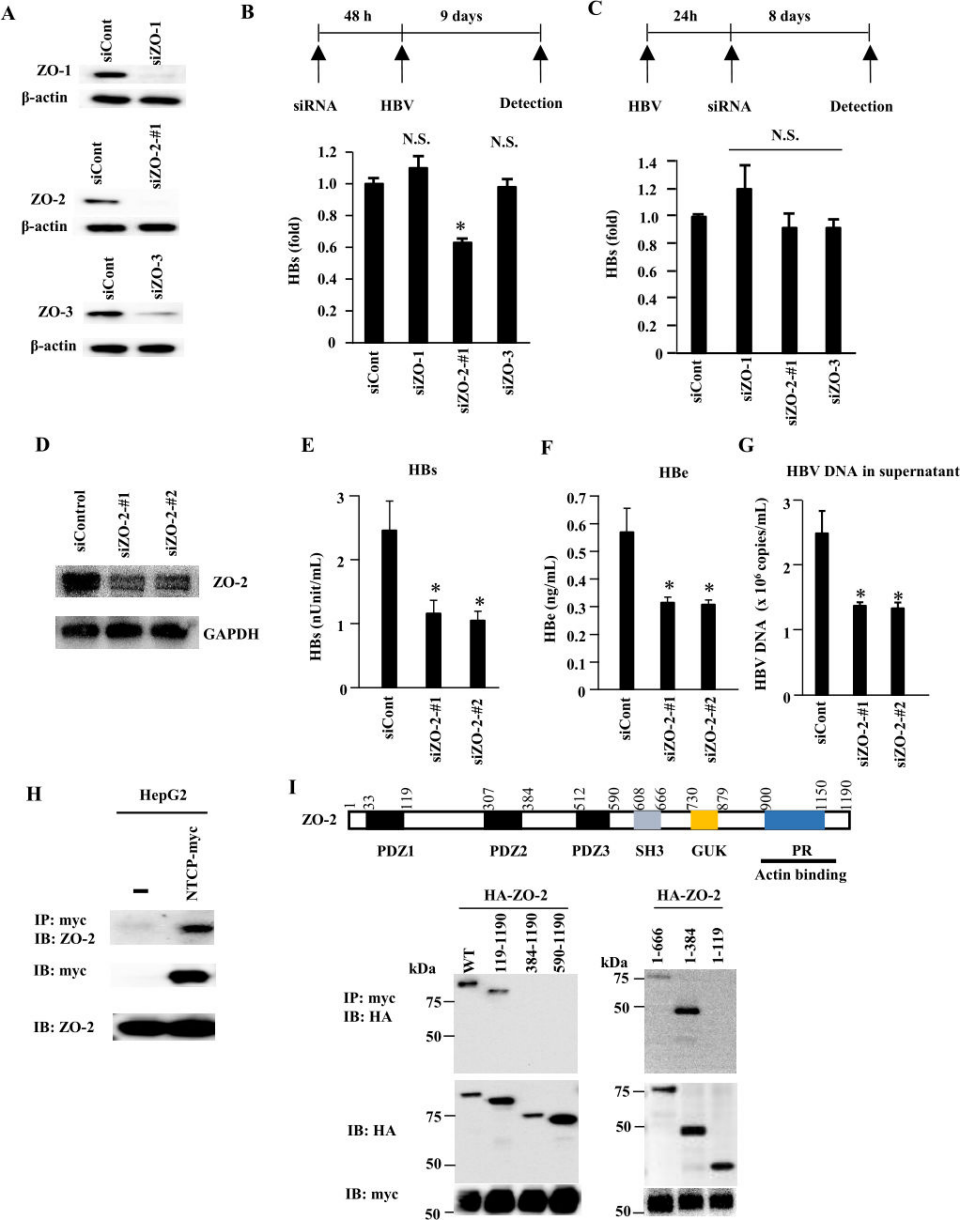
ZO-2, but not ZO-1 or ZO-3, supports the early stage of HBV infection, and the affinity for NTCP maps within the ZO-2 N-terminal half. (**A**) HepG2-NTCP cells were transfected with the indicated siRNA. At 48 h after transfection, cell lysates were analyzed by western blot using the indicated antibodies. (**B**) HepG2-NTCP cells were transfected with the indicated siRNA. Two days after transfection, cells were infected with HBV in the presence of 4% PEG8000 and 2% DMSO. The level of HBs was measured 9 days after infection. All graphs are presented as a mean ± s.d. (*n* = 3). ＊, *P* < 0.05; N.S., not significant. (**C**) HepG2-NTCP cells were infected with HBV in the presence of 4% PEG8000 and 2% DMSO. At 24 h after infection, cells were transfected with the indicated siRNA. The level of HBs was measured 8 days after transfection. All graphs are presented as a mean ± s.d. (*n* = 3). N.S., not significant. (**D**) Human primary hepatocytes were transfected with the indicated siRNA. Two days after the first transfection, cells were re-transfected with the indicated siRNA for 24 h. The levels of ZO-2 and GAPDH were determined by western blotting. (**E–G**) Human primary hepatocytes were transfected with the indicated siRNA. Two days after the first transfection, cells were re-transfected with the indicated siRNA for 24 h and then infected with HBV in the presence of 4% PEG8000 and 2% DMSO for 24 h. At 9 days after infection, HBs (**E**), HBe (**F**), and HBV DNA (**G**) levels in the culture supernatant were determined by ELISA and real-time PCR, respectively. All graphs are presented as a mean ± s.d. (*n* = 3). ＊, *P* < 0.05. (**H**) Cell extracts were subjected to immunoprecipitation with anti-myc antibody, followed by immunoblotting analysis with anti-ZO-2 antibody. (**I**) Full-length ZO-2 (1,190 amino acids) contains several distinct domains. HepG2-NTCP cells were transduced to express HA-tagged full-length (wild type) or truncated forms of ZO-2. The numbers indicate the amino acid regions represented by each expression construct. Cell lysates were subjected to immunoprecipitation using an anti-myc antibody, followed by immunoblotting with an anti-HA antibody.

Tight junctions (TJs) that connect cells in polarized epithelia are situated at the border between apical and basolateral membranes and contain a network of interacting proteins, including the related ZO-1, ZO-2, and ZO-3, which reside at the intracellular side of the plasma membrane (17–19). Notably, *in vivo* hepatocytes form polarized epithelia, but hepatocyte cell lines like HepG2 are inconsistent in their possible polarization and possession of tight junctions (20). Given our observations for ZO-2, by using siRNA experiments, we addressed whether ZO-1 or ZO-3 might have importance for HBV infection of HepG2-NTCP cells. While the knockdown of ZO-2 significantly decreased HBV infection, the knockdown of ZO-1 or ZO-3 had no notable effects (Fig. 3A and B). If the time order was changed, so that ZO-1, ZO-2, or ZO-3 were knocked down only after the first 24 h of HBV infection, none of the knockdowns had a significant impact on the level of HBsAg production 8 days after transfection of siRNA (Fig. 3C). Together, the data show that ZO-2 only has importance at the initial stage of HBV infection, while ZO-1 and ZO-3 are not important at any stage. To further validate the role of ZO-2 in HBV infection in primary human hepatocytes (PHHs), we performed siRNA-mediated knockdown of ZO-2 in PHHs (Fig. 3D). The silencing of ZO-2 significantly reduced the level of HBs (Fig. 3E), HBe (Fig. 3F), and extracellular HBV DNA (Fig. 3G).

The data also indicate that the importance of ZO-2 for HBV infection is independent of ZO-2 interactions with ZO-1 or ZO-3.

### Binding capacity for NTCP resides within the N-terminal half of ZO-2

Interaction between ZO-2 and NTCP was confirmed by anti-myc immunoprecipitation using lysates of HepG2-NTCP cells (which express myc-tagged NTCP), followed by ZO-2-specific western blot analysis (Fig. 3H). To map the part of ZO-2 responsible for the interaction with NTCP, several constructs for only expressing parts of ZO-2 were created. Full-length ZO-2 contains three PSD-95/DLG/ZO-1 (PDZ) domains, an Src homology 3 (SH3) domain, a region of homology to guanylate kinase referred to as the GUK domain, a proline-rich region, and an actin-binding region (Fig. 3I) (21). Expression constructs encoding N-terminal HA-tagged full-length ZO-2 and its truncated forms (amino acids 119–1190, 384–1190, 590–1190, 1–666, 1–384, and 1–119) were transduced into HepG2-NTCP cells. Anti-myc immunoprecipitation targeting NTCP-myc in cell extracts co-precipitated the full-length (WT) ZO-2, as well as its 119–1190, 1–666, and 1–384 amino acid variants, as detected by anti-HA immunoblotting (Fig. 3I). These results indicate that NTCP associates with the 119–384 aa stretch located within the N-terminal half of ZO-2.

### NTCP dissociates from ZO-2 upon preS1 peptide treatment or HBV infection

**Fig 4:**
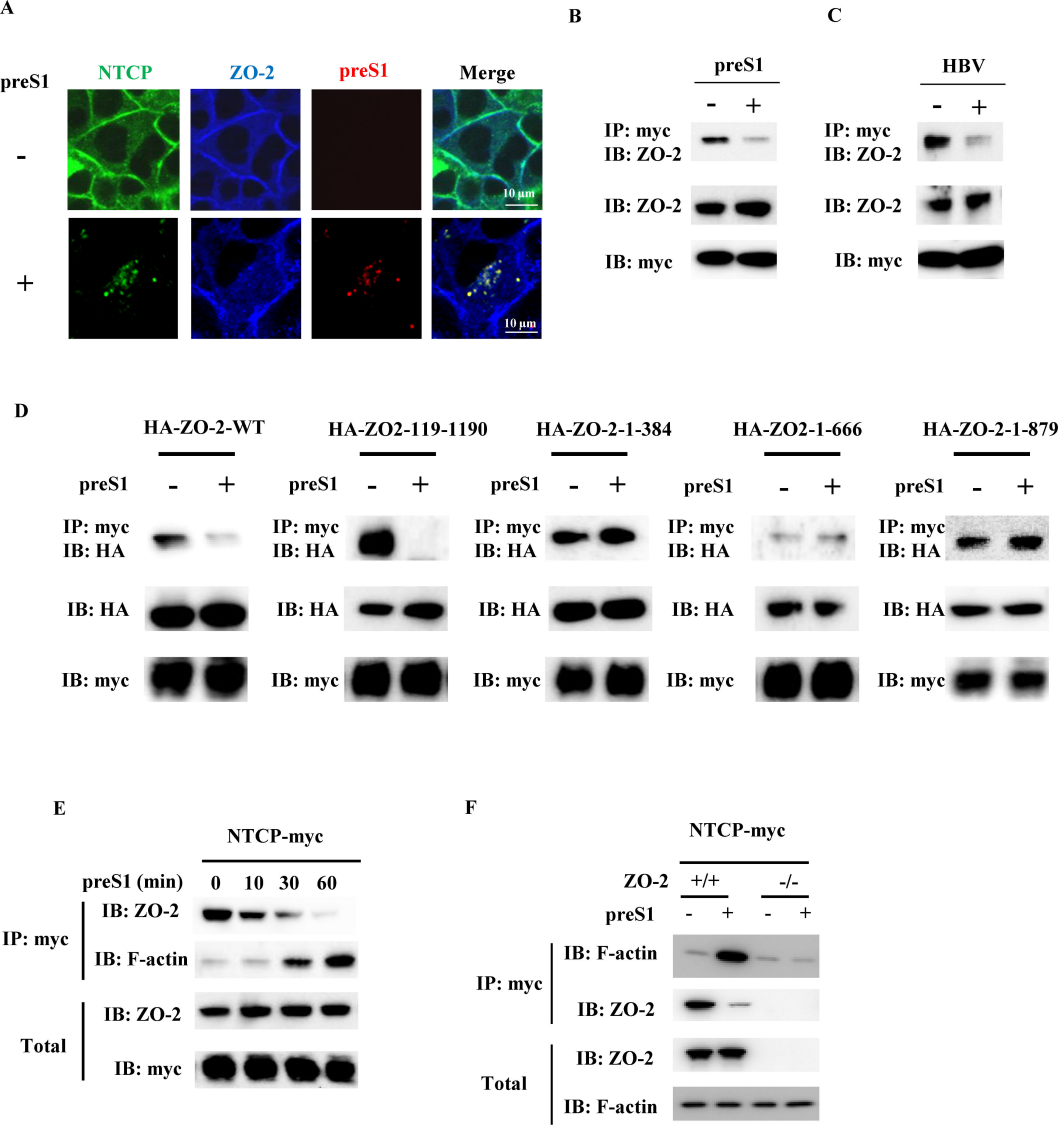
PreS1 treatment or HBV infection disrupts NTCP/ZO-2 interaction and leads to cell internalization of NTCP. (**A**) HepG2-NTCP cells were treated with 100 nM preS1-TAMRA (red) for 6 h. Cells were stained fluorescently for NTCP (green) and with 4′,6-diamidino-2-phenylindole (blue) and subjected to fluorescence microscopy. (**B and C**) HepG2-NTCP cells were treated with 100 nM preS1 peptide (**B**) or HBV (**C**) for 6 h. Cell lysates were subjected to immunoprecipitation with anti-myc antibody, followed by immunoblotting analysis with anti-ZO-2 antibody. (**D**) HepG2-NTCP cells were transduced with a lentiviral vector to express HA-tagged ZO-2 or its mutant variants. Cells were then treated with 100 nM preS1 peptide for 6 h. Cell lysates were subjected to immunoprecipitation using an anti-myc antibody, followed by immunoblotting with an anti-HA antibody. (**E**) HepG2-NTCP cells were treated with preS1 peptide for 10, 30, or 60 min. Cell lysates were subjected to immunoprecipitation with anti-myc antibody, followed by immunoblotting analysis with anti-ZO-2 antibody or anti-F-actin antibody. (**F**) Parental HepG2-NTCP cells (ZO-2^+^/^+^) and ZO-2⁻/⁻ HepG2-NTCP cells were treated with preS1 peptide for 4 h. Cell lysates were subjected to immunoprecipitation with anti-myc antibody, followed by immunoblotting analysis with anti-ZO-2 antibody or anti-F-actin antibody.

To further verify the association between NTCP and ZO-2 in cells, we examined the cellular localization of these two proteins in HepG2-NTCP cells after preS1 peptide treatment by using confocal microscopy (Fig. 4A). Immunofluorescence revealed that both NTCP and ZO-2 were strongly associated with the plasma membrane in the absence of preS1-TAMRA. Consistent with previous studies (15, 22), upon treatment with preS1 peptide for 6 h, NTCP was internalized along with preS1-TAMRA into cells as shown by intracellular speckles. Meanwhile, ZO-2 remained associated with the plasma membrane, suggesting that treatment with preS1-TAMRA disrupted NTCP/ZO-2 interaction. To further address the effect of preS1 peptide on NTCP/ZO-2 interaction, an immunoprecipitation assay was performed. HepG2-NTCP cells were treated with the preS1 peptide or mock-treated, and cell lysates were subsequently subjected to immunoprecipitation with an anti-myc antibody to assess the interaction between NTCP-myc and ZO-2. Precipitated immunocomplexes were analyzed by ZO-2-specific western blotting, revealing that treatment with preS1 peptide largely reduced the interaction of NTCP with ZO-2 (Fig. 4B); of note, preS1 peptide treatment had no notable effect on the total levels of ZO-2 and NTCP. Similar results were obtained after HBV infection (Fig. 4C) and taurocholic acid (Fig. S2).

To further identify the region of ZO-2 responsible for the preS1 peptide-induced reduction in NTCP/ZO-2 interaction, HepG2-NTCP cells were transduced with expression constructs encoding N-terminal HA-tagged ZO-2 fragments and subsequently treated with the preS1 peptide (Fig. 4D). Consistent with the result in Fig. 4B, preS1 peptide treatment led to a reduced NTCP/ZO-2 interaction, and such reduction was also observed for NTCP interaction with the 119–1,190 aa region of ZO-2. In contrast, there was no notable effect of preS1 treatment on the binding of NTCP to the 1–384 aa, 1–666 aa, and 1–879 aa regions of ZO-2. This suggests that the C-terminal region of ZO-2, which includes an actin-binding region, is necessary for the disruption of the NTCP/ZO-2 interaction initiated by preS1 peptide treatment or HBV infection.

To verify whether actin plays a role in the NTCP/ZO-2 interaction, an immunoprecipitation assay was performed. HepG2-NTCP cells were treated with preS1 peptide for 10–60 min. After treatment, cell lysates were subjected to immunoprecipitation with an anti-myc antibody, and the resulting immunocomplexes were analyzed by western blotting using anti-ZO-2 antibody and anti-F-actin antibody. The results show that the interaction of NTCP with ZO-2 rapidly decreased in the first 10 min after preS1 peptide treatment, and that NTCP was complexed with F-actin after 30-min treatment with preS1 peptide (Fig. 4E). Comparison between ZO-2^+/+^ HepG2-NTCP cells and ZO-2^-/-^ HepG2-NTCP cells indicated that in the absence of ZO-2, the preS1 treatment did not induce an interaction between NTCP and F-actin (Fig. 4F).

### Actin is required for HBV infection

**Fig 5:**
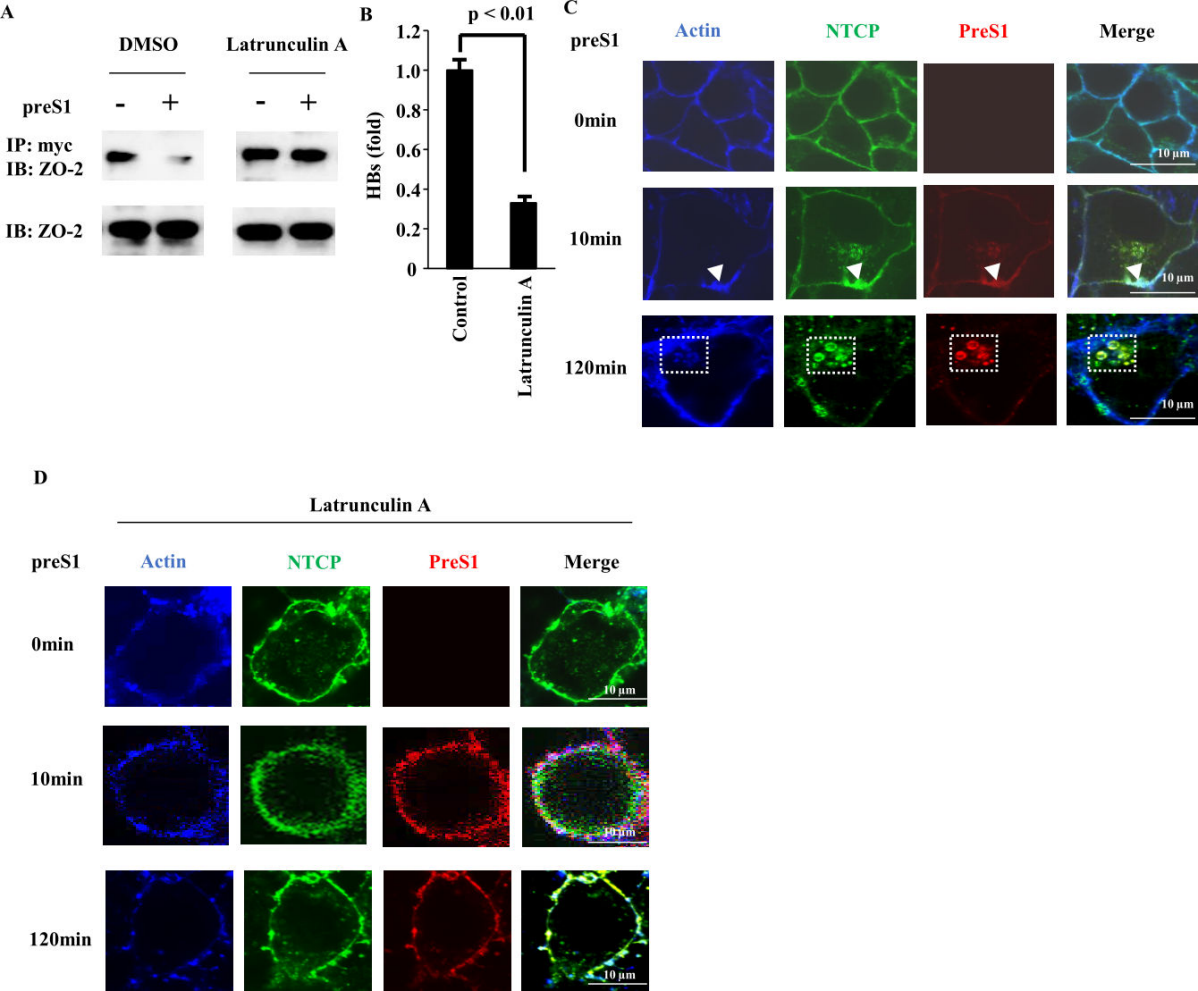
Latrunculin A blocks the internalization of HBV into the target cell. (**A**) HepG2-NTCP cells were treated with DMSO or 500 nM latrunculin A for 24 h and then treated with preS1 peptide for 6 h. Cell lysates were subjected to immunoprecipitation with anti-myc antibody, followed by immunoblotting analysis with anti-ZO-2 antibody. (**B**) HepG2-NTCP cells were treated with 500 nM latrunculin A for 24 h, followed by HBV infection in the presence of 4% PEG8000 and 2% DMSO. HBs levels were measured 9 days post-infection. All graphs are presented as a mean ± s.d. (*n* = 3). (**C**) HepG2-NTCP cells were treated with 100 nM preS1-TAMRA (red) for 10 or 120 min. Cells were stained fluorescently for NTCP (green) and with 4′,6-diamidino-2-phenylindole (DAPI) (blue) and subjected to fluorescence microscopy. (**D**) HepG2-NTCP cells were treated with 500 nM latrunculin A. At 24 h after treatment, cells were incubated with 100 nM preS1-TAMRA (red) for 10 or 120 min. Cells were stained fluorescently for NTCP (green) and with DAPI (blue) and subjected to fluorescence microscopy.

To further investigate the functional impact of actin, we used latrunculin A to inhibit actin polymerization. HepG2-NTCP cells were treated with latrunculin A in DMSO or with DMSO control. At 24 h after treatment, cells were incubated with preS1 peptide in DMSO or with DMSO control for 6 h. Cell lysates were then subjected to immunoprecipitation using anti-myc antibody for targeting NTCP-myc. Western blot analysis of complexes immunoprecipitated with an anti-myc antibody, using an anti-ZO-2 antibody, revealed that the preS1-induced reduction in NTCP/ZO-2 interaction (Fig. 5A, left) was abolished by treatment with latrunculin A (Fig. 5A, right). We next assessed whether latrunculin A affects HBV infection. HepG2-NTCP cells were treated with latrunculin A in DMSO or with DMSO control for 24 h and then infected with HBV. Latrunculin A significantly inhibited HBV infection (Fig. 5B), suggesting that actin polymerization is required for HBV infection. This agrees with recent findings by Herrscher et al. (10).

To assess the involvement of actin during HBV entry, immunofluorescence analysis was performed using confocal microscopy (Fig. 5C). In the absence of preS1-TAMRA, actin (stained by Alexa405-phallodine) mainly localized at the plasma membrane. After 10 min of treatment, fluorescence patterns suggested the presence of NTCP-preS1-actin complexes near the cell surface, and after 120 min of treatment, such complexes were consistently found intracellularly in what seemed to be vesicular structures. However, the internalization of NTCP, preS1-TAMRA, and actin was not observed in cells treated with latrunculin A (Fig. 5D). Taken together, these results indicate that ZO-2 regulates the membrane localization of NTCP and transfers the NTCP-preS1 complex to actin-associated structures to support HBV infection.

## DISCUSSION

In this study, ZO-2 was identified as a binding partner of NTCP (Table 1; Fig. 3H and I). ZO-2 was initially isolated as a ZO-1-interacting protein in epithelial cells (23), but we did not find an interaction of ZO-1 with NTCP in our immunopurification assay (Table 1). ZO-2 largely enhanced the cell surface localization of NTCP (Fig. 2C and D), stabilized NTCP protein (Fig. 2B), and enabled HBV to efficiently enter hepatocytes (Fig. 2G through J). PreS1 peptide treatment or HBV infection triggered the disruption of NTCP/ZO-2 interaction (Fig. 4B and C), a process that required the actin-binding region of ZO-2 (Fig. 4D). Importantly, preS1 peptide treatment induced the association of NTCP with F-actin (Fig. 4E).

At the cell membrane, ZO-2 concentrates at tight junctions (24) and—at least in some cell types—also at adherens junctions (AJs) (25). TJs in epithelial cells mediate cell-cell adhesion and act as a barrier to intramembrane diffusion between apical and basolateral membrane domains (26), while AJs, which also mediate cell-cell adhesion, are positioned below TJs (27). The scaffold protein ZO-2 is not located in membranes but binds transmembrane molecules such as occludin and claudins, as well as membrane-associated molecules such as α-catenin, and links them with other junction proteins and the actin cytoskeleton (25, 28). Other than at TJs and AJs, the ZO-2 distribution at the cell membrane has not been rigorously investigated. A previous report indicated that ZO-2 distribution may be interpreted as ZO-2 accumulating at other membrane locations before forming a sink to concentrate transmembrane proteins that build cell junction complexes (29). ZO-2 stabilizes membrane proteins and membrane organization, as exemplified by ZO-2 silencing causing lower amounts of E-cadherin, occludin (30), claudin-7, and integrin β1 (31), and exclusion of E-cadherin from the basolateral membrane (30). In sparse cell cultures, ZO-2 concentrates in the nucleus (32), and ZO-2 can have a profound effect on the cell transcriptome (28). However, in our cell system, we did not observe ZO-2 in the nuclei, and a transcription function of ZO-2 is unlikely to be responsible for the observed effects of ZO-2 knockout on NTCP distribution (Fig. 2C and D) and stability (Fig. 2B).

NTCP is a transmembrane glycoprotein expressed at the basolateral membrane of hepatocytes, where it mediates uptake of bile acids from the sinusoids (33) and infection by HBV (34). A large part of NTCP was shown to reside in lipid rafts (detergent-resistant membrane fractions) (35), but not much is known about the precise location of NTCP within the basolateral membrane. Recently, Hu et al. (36) found that NTCP was associated with E-cadherin in an immunopurification assay. E-cadherin is a major constituent of AJs (37), but some E-cadherin can also be found outside AJs (38, 39). Hu et al. (36) found that knockdown of E-cadherin led to a ~90% decrease in HBV infection, despite the fact that the amounts of NTCP at the cell surface were only slightly decreased. The combination of our data with those of Hu et al. (36) suggests that ZO-2 stabilizes NTCP at the cell surface, but that E-cadherin is necessary to turn cell surface NTCP into an efficient receptor for HBV, possibly by associating NTCP/ZO-2 complexes with AJs.

Our findings that latrunculin A, an inhibitor of actin polymerization, interfered with HBV infection (Fig. 5B) and preS1 internalization (Fig. 5D) agree with previous studies showing that HBV entry into HepG2‐NTCP and other hepatocytes involves clathrin‐mediated endocytosis (CME) (10, 11). We interpret the associations of NTCP and actin after preS1 stimulation (Fig. 4E and F) as evidence for NTCP-preS1 complexes having entered the CME route and acknowledge that the co-isolation of actin with NTCP does not necessarily indicate their direct binding to each other but could represent larger CME complexes.

**Fig 6:**
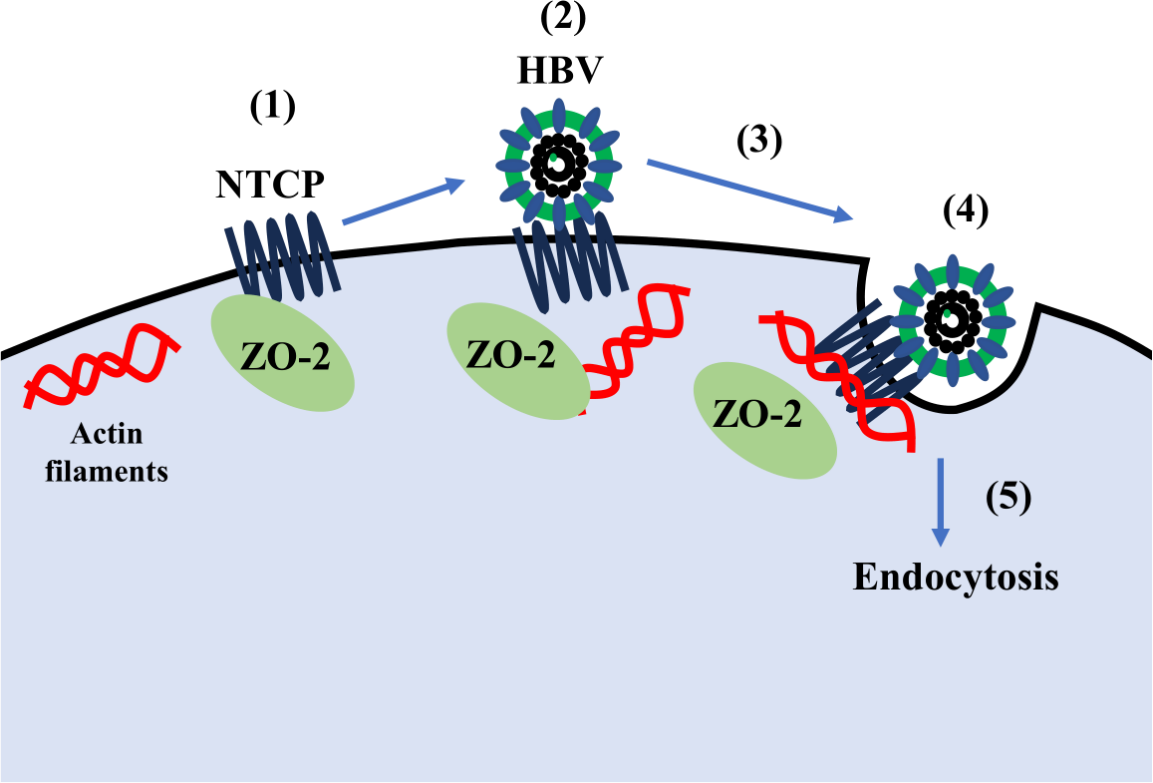
A schematic model depicting the role of ZO-2 in HBV internalization mediated by NTCP. (1) ZO-2 stabilizes NTCP at the cell membrane; (2) upon HBV binding, NTCP or NTCP/ZO-2 complexes are directed to CME sites; (3) entry via NTCP requires disruption of the ZO-2–NTCP interaction; (4) this disruption depends on the actin-binding region of ZO-2; (5) possibly by facilitating the delivery of NTCP/ZO-2 complexes to CME sites.

Based on our data together with the above considerations, we propose the following model for the involvement of ZO-2 in NTCP-mediated infection of HBV to hepatocytes (Fig. 6). ZO-2 localizes and stabilizes NTCP at the cell membrane. Upon HBV binding to NTCP, ZO-2 dissociates from NTCP, allowing NTCP to associate with actin and facilitate HBV internalization. In the future, more detailed studies of NTCP on membrane localizations are necessary to test this model, besides the important roles of ZO-2 described here.

Our findings are also relevant for understanding a disease phenotype. NTCP plays a crucial role in maintaining bile acid homeostasis by mediating their uptake from the bloodstream into hepatocytes (33). Progressive familial intrahepatic cholestasis (PFIC) is a human disease characterized by an impaired circulation of bile acids and their elevated level in the serum (40). In a subset of PFIC patients, homozygous mutations in *ZO-2* were identified as the cause of this severe disease (41). PFIC caused by absent ZO-2 function may be explained by a disturbance of the integrity of the hepatocyte epithelium through a loss of proper intercellular junctions (41), or by the absence of localization of NTCP at the cell membrane, as demonstrated in the present study. Interestingly, a study showed that in livers of PFIC patients with other genetic causes (PFIC-2 and -3 types), the level of NTCP protein but not mRNA was lower than in healthy controls (42), suggesting that a reduction of NTCP may be a common explanation for the decreased uptake of bile acid in PFIC disease. Therefore, based on our results, it might be considered whether manipulation of ZO-2 could benefit PFIC patients.

Given that NTCP is already a clinically approved target for the treatment of chronic hepatitis D via the entry inhibitor bulevirtide, our results may have therapeutic implications. Specifically, modulation of ZO-2 function could represent an alternative or complementary strategy to control NTCP-mediated HBV entry. Targeting host factors such as ZO-2 may offer a novel approach to indirectly regulate the localization of NTCP at the cell surface, potentially expanding the antiviral drug against HBV and HDV.

In summary, our study contributed to the elucidation of ZO-2 function, NTCP cellular distribution, and the HBV infection route, and hopefully may help in the development of therapies against PFIC.

## MATERIALS AND METHODS

### Cells

HepG2 cells were maintained at 37°C and 5% CO_2_ in Dulbecco’s modified Eagle’s medium (DMEM) (Thermo Fisher Scientific) supplemented with 10% fetal bovine serum (FBS), 100 U/mL penicillin, 100 μg/mL streptomycin (Nacalai Tesque), and 100 U/mL nonessential amino acids (Thermo Fisher Scientific). HepAD38 cells were maintained at 37°C and 5% CO_2_ in DMEM–F-12 GlutaMAX (Thermo Fisher Scientific) supplemented with 10% FBS, 100 U/mL penicillin, 100 μg/mL streptomycin, 5 μg/mL insulin (Sigma-Aldrich), and 500 ng/mL tetracycline (TAKARA Bio).

Primary human hepatocytes (PXB cells) were purchased from PhoenixBio and cultured at 37°C and 5% CO_2_ in hepatocyte clonal growth medium (PhoenixBio).

To establish HepG2-NTCP cells, HepG2 cells were transfected with pCAN-NTCP-myc using Lipofectamine 3000 (Thermo Fisher Scientific) according to the manufacturer’s instructions. At 2 days after transfection, cells were treated with 500 μg/mL G418 (Thermo Fisher Scientific) to select a stable cell line that showed the highest susceptibility to HBV infection among the established subclones. HepG2-NTCP cells were maintained in DMEM supplemented with 10% FBS, 100 U/mL penicillin, 100 μg/mL streptomycin, 100 U/mL nonessential amino acids, and 500 μg/mL G418.

To establish ZO-2^-/-^ HepG2-NTCP cells, HepG2-NTCP cells were transduced with Cas9 along with human ZO-2-targeting sgRNA using the lentivirus vector. Cells were treated with 1.5 μg/mL puromycin, and single-cell colonies were collected. The knockout of ZO-2 was confirmed by western blotting and sequencing of the target region.

To create HepG2-HA-NTCP-myc cells (expressing HA- and myc-tagged NTCP), which were only used for proteomics experiments, HepG2 cells were transduced with HA-NTCP-myc using a lentiviral vector system.

### Reagents

Reagents and, in the case of antibodies, their concentrations are described in Table S1.

### Plasmid

To express C-terminal myc-tagged NTCP (NTCP-myc) using the CAG promoter in HepG2 cells, pCAN-NTCP-myc was constructed as described previously (43).

To construct a lentiviral vector encoding N-terminal HA- and C-terminal myc-tagged NTCP (HA-NTCP-myc), the HA-NTCP-myc fragment was inserted into the EcoRI and BamHI sites of CSII-CMV-MCS using an In-Fusion HD Cloning Kit (TAKARA Bio) according to the manufacturer’s instructions. HA-tagged ZO-2 fragments corresponding to amino acids 119–1,190, 1–384, 1–666, and 1–879 were inserted into the EcoRI and BamHI sites of the CSII-CMV-MCS vector using the In-Fusion HD Cloning Kit. The pLenti-EF1a-Cas9-Puro encoded Cas9 nuclease was purchased from Applied Biological Materials. To express human ZO-2-targeting sgRNA (target sequence: CGTAGTCCTGGTCAATGCTC), pLenti-U6-sgRNA-PGK-Neo was purchased from Applied Biological Materials (Cat#: 467451110395). For the lentiviral vector system, pCAG-HIVgp, pCMV-VSV-G, and pRSV-Rev were kindly provided by Dr. Hiroyuki Miyoshi at RIKEN.

### Measurement of HBsAg and HBeAg

HepG2-NTCP cells were infected with HBV in the presence of 4% PEG8000 and 2% DMSO for 9 days. The level of HBs in the culture supernatant was determined by Lumipulse assay (Fujirebio) or HBs Antigen Quantitative ELISA Kit, Rapid-2 (Beacle) according to the manufacturer’s protocol. The level of HBe in the culture supernatant was determined by QuickTiter Hepatitis B “e” Antigen (HBeAg) ELISA Kit (Cell Biolabs) according to the manufacturer’s protocol.

### Immunofluorescence analysis

HepG2-NTCP cells were cultured in 8-well chambers coated with rat tail collagen type I (Merck Millipore) at 37°C for 24 h. Cells were treated with 100 nM preS1-TAMRA at 4°C for 1 h and then washed with PBS. The preS1-treated cells were cultured at 37°C to allow internalization for 10 min, 120 min, or 6 h. Cells were fixed with 4% paraformaldehyde and permeabilized with 0.3% Triton X-100. Cells were then stained with primary antibodies for 2 h at room temperature. After cells were washed with PBS, they were incubated with Alexa-conjugated secondary antibodies (Thermo Fisher Scientific) or Alexa405-conjugated phalloidin (Thermo Fisher Scientific) at room temperature for 1 h. The nucleus was stained with 4′,6-diamidino-2-phenylindole. The stained cells were observed by laser-scanning confocal microscopy using a FluoView FV1000 and fluorescence microscopy using an IX73 (Olympus).

### HBV attachment assay

HepG2 cells and HepG2-NTCP cells were plated at 2 × 10^5^ cells/well in a 24-well plate. At 24 h after plating, cells were incubated with 1,000 genome equivalents (GEq)/cell HBV at 4°C for 3 h and then washed to remove free HBV. DNA of cells plus cell-surface HBV was extracted by DNeasy Blood & Tissue Kits (QIAGEN), and the HBV DNA was quantified by real-time PCR using Fast SYBR Green Master Mix (Thermo Fisher Scientific) and the Fast Real‐Time PCR system (Thermo Fisher Scientific). The primers used were 5′‐CTCGTGGTGGACTTCTCT‐3′ (forward) and 5′‐AAGATGAGGCATAGCAGCA-3′ (reverse). HBV attachment involving NTCP was calculated by subtracting the level of HBV DNA observed for HepG2 cells (as the background level) from the level of HBV DNA observed for HepG2-NTCP cells.

### HBV infection

To obtain HBV genotype D, HepAD38 cells were cultured with tetracycline-free medium. Virus particles were concentrated by precipitation with PEG8000 (13% PEG8000, 1.5 M NaCl, 1 mM EDTA [pH 8.0], and 10 mM HEPES [pH 7.6]). After PEG precipitation, the virus was further purified by precipitation through 20% sucrose in TNE (10 mM Tris-HCl, 50 mM NaCl, and 1 mM EDTA) at 100,000 × *g* for 3 h. The virus was then suspended in Opti‐MEM (Thermo Fisher Scientific) and stored at −80°C until use. HepG2-NTCP cells or human primary hepatocytes were infected with HBV at 5,000 or 100 GEq/cell, respectively, in the presence of 4% PEG8000 and 2% DMSO.

### siRNA

siRNAs were transfected into HepG2-NTCP cells using Lipofectamine RNAiMAX (Thermo Fisher Scientific) according to the manufacturer’s protocol. Negative control siRNA was purchased from Integrated DNA Technologies (Cat# 51-01-14-04). Target sequences of siZO-1, siZO-2, siZO-3, siSDC4, siTFRC, siSTT3A, and siSTT3B are as follows:

siZO-1, 5′-ACGCTATTGAATGTCCCTGATCTTTCT-3′;

siZO-2-#1, 5′-ACCTTTTTACAGCTACAATCAACCT-3′;

siZO-2-#2, 5′-GTTTGGCAGCTTAAAGGACACTATT-3′;

siZO-3, 5′-TCAGATACTCAAGACCTGCACCAAGAT-3′;

siSDC4, 5′-ACCAATGAGTTCTACGCGTGAAGCTTG-3′;

siTFRC, 5′-CTGTGAATGGATCTATAGTGATTGTCA-3′;

siSTT3A, 5′-GAGTTTGATCCGTACTTTAATTATCGG-3′;

siSTT3B, 5′-TTGAACATAACTGTTCACATAAGAGAC-3′.

### RT-qPCR

Total RNA from HepG2-NTCP cells was extracted with RNeasy Mini Kit (QIAGEN) according to the manufacturer’s protocol and was reverse transcribed into complementary DNA using ReverTra Ace qPCR RT Master Mix with gDNA Remover (TOYOBO). qPCR was carried out with PowerUp SYBR Green master mix (Thermo Fisher Scientific), and fluorescence was analyzed with StepOnePlus real-time PCR system (Thermo Fisher Scientific). Primer sequences of STT3A, STT3B, Sec61A1, SDC4, TFRC, and ZO-2 are as follows:

STT3A-F, 5′-GAAGCAACAGGATTCCACCTACC-3′;

STT3A-R, 5′-CAATGGACGGAGAAGAGTAGGC-3′;

STT3B-F, 5′-AAGTGAACACATGGCAGCTG-3′;

STT3B-R, 5′-TCGGTCTCTCAGATACTGCAA-3′;

Sec61A1-F, 5′-CTCGCTTCAGTGGCAACTTGCT-3′;

Sec61A1-R, 5′-GCCACCAACTGGATAAGCACGT-3′;

SDC4-F, 5′-GAGTGAGGATGTGTCCAACAAGG-3′;

SDC4-R, 5′-GGTACATGAGCAGTAGGATCAGG-3′;

TFRC-F, 5′-ATCGGTTGGTGCCACTGAATGG-3′;

TFRC-R, 5′-ACAACAGTGGGCTGGCAGAAAC-3′;

ZO-2-F, 5′-ATTAGTGCGGGAGGATGCCGTT-3′;

ZO-2-R, 5′-TCTGCCACAAGCCAGGATGTCT-3′.

### cccDNA analysis

Extraction of cccDNA was performed using Hirt extraction. Briefly, infected cells were suspended in 1.5 mL of lysis buffer (10 mM Tris-HCl [pH 7.5], 10 mM EDTA, and 0.7% SDS) and incubated at room temperature for 30 min. Subsequently, 0.4 mL of 5 M NaCl was added to the lysate, and the mixture was incubated overnight at 4°C. The lysate was clarified by centrifugation at 12,000 × *g* for 30 min at 4°C. DNA was purified from the supernatant using the DNeasy Blood & Tissue Kits (QIAGEN) and treated with T5 Exonuclease (New England Biolabs) to remove rcDNA. Quantification of cccDNA was performed by qPCR using a cccDNA-specific primer set: cccDNA-probe, 5′-[FAM]CTGTAGGCATAAATTGGT[MGBEQ]−3′; HBV-cccDNA-F, 5′-CGTCTGTGCCTTCTCATCTGC-3′; and HBV-cccDNA-R, 5′-GCACAGCTTGGAGGCTTGAA-3′.

### Preparation of lentiviral vector

HEK293T cells were transfected with pCAG-HIVgp, pCMV-VSV-G, and pRSV-Rev along with the transfer vector by using Lipofectamine3000 according to the manufacturer’s protocol. At 2 days after transfection, culture supernatants were collected and filtered through 0.45-μm-pore-size filters (Millipore).

### Immunoprecipitation

HepG2-NTCP cells (expressing myc-tagged NTCP) were lysed with IP Lysis buffer (Thermo Fisher Scientific) containing protease inhibitor cocktail tablets (Roche). Extracts were incubated with c-Myc tagged Protein Mild Purification Gel (Medical & Biological Laboratories) at 4°C for 90 min. After incubation, the beads were washed with IP lysis buffer and then eluted with 250 µg/mL 3× myc peptide (Medical & Biological Laboratories). The purified immunocomplexes were resolved by SDS-PAGE and transferred onto nitrocellulose membranes, which were blocked with 5% skim milk. ZO-2 or HA was detected with anti-ZO-2 antibody (Thermo Fisher Scientific) or anti-HA antibody (Cell Signaling Technology), respectively. A horseradish peroxidase-conjugated anti-rabbit IgG antibody (GE Healthcare Life Sciences) was used as the secondary antibody.

### Proteomic analysis

HepG2-HA-NTCP-myc cells (expressing HA- and myc-tagged NTCP) were lysed in IP Lysis buffer containing protease inhibitor cocktail tablets. HA-NTCP-myc was precipitated with c-Myc tagged Protein Mild Purification Gel and eluted with 250 µg/mL 3× myc peptide. Eluates were further incubated with anti-HA magnetic beads. The beads were washed six times with IP buffer and eluted with 250 µg/mL HA peptide (Medical & Biological Laboratories). Five percent of the purified immunocomplex was resolved on SDS-PAGE and stained with the SimplyBlue SafeStain (Thermo Fisher Scientific). To the remaining eluate, trichloroacetic acid was added to a final concentration of 10%. The samples were incubated on ice for 30 min and centrifuged at 18,800 × *g* at 4°C for 30 min. The resulting pellet was resuspended in 500 µL of ice-cold acetone and then centrifuged at 18,800 × *g* at 4°C for 30 min. The pellet was then washed two times with 500 µL of ice-cold acetone and finally resuspended in buffer (50 mM TEAB and 0.1% SDS). A total of 2 µg of protein was reduced by adding DTT to a final concentration of 134 mM and incubating at 35°C for 2 h. Subsequently, free thiol groups were alkylated with 230 mM IAA at room temperature for 30 min in the dark. Samples were digested with trypsin overnight at 37°C. The recovered peptides were analyzed using Q Exactive Plus (Thermo Fisher Scientific) coupled online with a capillary high-performance liquid chromatography system (EASY-nLC 1200, Thermo Fisher Scientific) to acquire MS/MS spectra. A 0.075 × 150 mm-EASY-Spray column (3 μm particle diameter, 100 Å pore size, Thermo Fisher Scientific) with mobile phases of 0.1% formic acid and 0.1% formic acid/80% acetonitrile was used. Data derived from the MS/MS spectra were used to search the protein database SWISS-Prot using the MASCOT Server (Matrix Science) and to identify proteins using the program Scaffold viewer (Proteome Software); all proteins identified with a calculated probability of ≥95% are listed in Table 1.

### Flow cytometry

To detect cell surface NTCP, cells were incubated with anti-NTCP antibody in staining buffer (2% BSA and 2 mM EDTA in PBS) on ice for 1 h. Cells were washed with staining buffer and then incubated with Alexa Fluor 488 conjugated anti-mouse IgG antibody in staining buffer on ice for 1 h. Cells were washed, and dead cells were distinguished by staining with 0.5 µg/mL of 7-AAD. Alexa Fluor 488 staining of living cells was analyzed using FACS Calibur (BD Biosciences) and Cell Quest software.

To assess preS1 binding, cells were incubated with preS1-TAMRA on ice for 1 h. Cells were washed with PBS and then analyzed using FACS Calibur (BD Biosciences) and Cell Quest software.

### Production of a monoclonal antibody against NTCP

A mouse monoclonal antibody that specifically recognized NTCP was generated using the DNA immunization and medial iliac lymph node method (44). Three B6D2F1/Slc mice were immunized once with the pDNAimmu3-human NTCP plasmid by electroporation, and one boost was given by injection of NTCP-overexpressing HEK293T cells at the tail base 12 weeks after immunization. Four days after the boost, the cells from spleens and medial iliac lymph nodes of mice immunized with the antigen were fused with mouse myeloma SP2 cells at a ratio of 5:1 in a polyethylene glycol solution (PEG1500; Life Technologies). The resulting hybridoma cells were plated onto 96-well plates and cultured in HAT selection medium (Hybridoma-SFM [Life Technologies], 10% FBS, 1 ng/mL mouse IL-6 [R&D Systems], 100 mM hypoxanthine [Sigma], 0.4 mM aminopterin [Sigma], and 16 mM thymidine [Wako]). At 8 days post-fusion, the hybridoma supernatants were screened using immunofluorescence. Finally, a hybridoma clone producing a monoclonal antibody named 5A9C6 was selected. The MAb 5A9C6 was found to be an IgG 1 (k) subtype using a mouse isotyping kit and reacts with the extracellular N-terminal domain of NTCP.

### Statistical analysis

The statistical significance of differences between two groups was evaluated by a two-tailed unpaired *t*-test. *P* < 0.05 was considered statistically significant.

## SUPPLEMENTAL MATERIAL

Fig. S1 mbio.02704-25-s0001.tiff Knockdown efficiency of the indicated siRNA in HepG2-NTCP cells.

Fig. S2 mbio.02704-25-s0002.tiff TCA treatment disrupts NTCP/ZO-2 interaction.

Table S1 mbio.02704-25-s0003.tiff Reagents.

ASM does not own the copyrights to Supplemental Material that may be linked to, or accessed through, an article. The authors have granted ASM a non-exclusive, world-wide license to publish the Supplemental Material files. Please contact the corresponding author directly for reuse.

## References

[1] Trépo C, Chan HLY, Lok A. 2014. Hepatitis B virus infection. Lancet 384:2053–2063. doi:10.1016/S0140-6736(14)60220-8 24954675

[2] Sureau C, Salisse J. 2013. A conformational heparan sulfate binding site essential to infectivity overlaps with the conserved hepatitis B virus a-determinant. Hepatology 57:985–994. doi:10.1002/hep.26125 23161433

[3] Qiao L, Luo GG. 2019. Human apolipoprotein E promotes hepatitis B virus infection and production. PLoS Pathog 15:e1007874. doi:10.1371/journal.ppat.1007874 31393946 PMC6687101

[4] Blanchet M, Sureau C. 2007. Infectivity determinants of the hepatitis B virus pre-S domain are confined to the N-terminal 75 amino acid residues. J Virol 81:5841–5849. doi:10.1128/JVI.00096-07 17376925 PMC1900317

[5] Meier A, Mehrle S, Weiss TS, Mier W, Urban S. 2013. Myristoylated PreS1-domain of the hepatitis B virus L-protein mediates specific binding to differentiated hepatocytes. Hepatology 58:31–42. doi:10.1002/hep.26181 23213046

[6] Yan H, Zhong G, Xu G, He W, Jing Z, Gao Z, Huang Y, Qi Y, Peng B, Wang H, Fu L, Song M, Chen P, Gao W, Ren B, Sun Y, Cai T, Feng X, Sui J, Li W. 2012. Sodium taurocholate cotransporting polypeptide is a functional receptor for human hepatitis B and D virus. eLife 3:e00049. doi:10.7554/eLife.00049 PMC3485615 23150796

[7] He W, Ren B, Mao F, Jing Z, Li Y, Liu Y, Peng B, Yan H, Qi Y, Sun Y, Guo JT, Sui J, Wang F, Li W. 2015. Hepatitis D virus infection of mice expressing human sodium taurocholate co-transporting polypeptide. PLoS Pathog 11:e1004840. doi:10.1371/journal.ppat.1004840 25902143 PMC4406467

[8] He W, Cao Z, Mao F, Ren B, Li Y, Li D, Li H, Peng B, Yan H, Qi Y, Sun Y, Wang F, Sui J, Li W. 2016. Modification of three amino acids in sodium taurocholate cotransporting polypeptide renders mice susceptible to infection with hepatitis D virus in vivo. J Virol 90:8866–8874. doi:10.1128/JVI.00901-16 27466423 PMC5021397

[9] Li W, Urban S. 2016. Entry of hepatitis B and hepatitis D virus into hepatocytes: basic insights and clinical implications. J Hepatol 64:S32–S40. doi:10.1016/j.jhep.2016.02.011 27084034 PMC7114860

[10] Herrscher C, Pastor F, Burlaud-Gaillard J, Dumans A, Seigneuret F, Moreau A, Patient R, Eymieux S, de Rocquigny H, Hourioux C, Roingeard P, Blanchard E. 2020. Hepatitis B virus entry into HepG2-NTCP cells requires clathrin-mediated endocytosis. Cell Microbiol 22:e13205. doi:10.1111/cmi.13205 32216005

[11] Huang HC, Chen CC, Chang WC, Tao MH, Huang C. 2012. Entry of hepatitis B virus into immortalized human primary hepatocytes by clathrin-dependent endocytosis. J Virol 86:9443–9453. doi:10.1128/JVI.00873-12 22740403 PMC3416113

[12] Diallinas G, Martzoukou O. 2019. Transporter membrane traffic and function: lessons from a mould. FEBS J 286:4861–4875. doi:10.1111/febs.15078 31583839

[13] Bijsmans I, Bouwmeester RAM, Geyer J, Faber KN, van de Graaf SFJ. 2012. Homo- and hetero-dimeric architecture of the human liver Na⁺-dependent taurocholate co-transporting protein. Biochem J 441:1007–1015. doi:10.1042/BJ20111234 22029531

[14] Fukano K, Tsukuda S, Oshima M, Suzuki R, Aizaki H, Ohki M, Park SY, Muramatsu M, Wakita T, Sureau C, Ogasawara Y, Watashi K. 2018. Troglitazone impedes the oligomerization of sodium taurocholate cotransporting polypeptide and entry of hepatitis B virus into hepatocytes. Front Microbiol 9:3257. doi:10.3389/fmicb.2018.03257 30671048 PMC6331526

[15] Iwamoto M, Saso W, Sugiyama R, Ishii K, Ohki M, Nagamori S, Suzuki R, Aizaki H, Ryo A, Yun JH, Park SY, Ohtani N, Muramatsu M, Iwami S, Tanaka Y, Sureau C, Wakita T, Watashi K. 2019. Epidermal growth factor receptor is a host-entry cofactor triggering hepatitis B virus internalization. Proc Natl Acad Sci USA 116:8487–8492. doi:10.1073/pnas.1811064116 30952782 PMC6486715

[16] Appelman MD, Chakraborty A, Protzer U, McKeating JA, van de Graaf SFJ. 2017. N-Glycosylation of the Na+-taurocholate cotransporting polypeptide (NTCP) determines its trafficking and stability and is required for hepatitis B virus infection. PLoS One 12:e0170419. doi:10.1371/journal.pone.0170419 28125599 PMC5268470

[17] Zihni C, Mills C, Matter K, Balda MS. 2016. Tight junctions: from simple barriers to multifunctional molecular gates. Nat Rev Mol Cell Biol 17:564–580. doi:10.1038/nrm.2016.80 27353478

[18] Stevenson BR, Siliciano JD, Mooseker MS, Goodenough DA. 1986. Identification of ZO-1: a high molecular weight polypeptide associated with the tight junction (zonula occludens) in a variety of epithelia. J Cell Biol 103:755–766. doi:10.1083/jcb.103.3.755 3528172 PMC2114282

[19] Itoh M, Furuse M, Morita K, Kubota K, Saitou M, Tsukita S. 1999. Direct binding of three tight junction-associated MAGUKs, ZO-1, ZO-2, and ZO-3, with the COOH termini of claudins. J Cell Biol 147:1351–1363. doi:10.1083/jcb.147.6.1351 10601346 PMC2168087

[20] Théard D, Steiner M, Kalicharan D, Hoekstra D, van Ijzendoorn SCD. 2007. Cell polarity development and protein trafficking in hepatocytes lacking E-cadherin/beta-catenin-based adherens junctions. Mol Biol Cell 18:2313–2321. doi:10.1091/mbc.e06-11-1040 17429067 PMC1877101

[21] Gonzalez-Mariscal L, Bautista P, Lechuga S, Quiros M. 2012. ZO-2, a tight junction scaffold protein involved in the regulation of cell proliferation and apoptosis. Ann N Y Acad Sci 1257:133–141. doi:10.1111/j.1749-6632.2012.06537.x 22671599

[22] Oshima M, Stappenbeck F, Ohashi H, Iwamoto M, Fukano K, Kusunoki A, Zheng X, Wang F, Morishita R, Aizaki H, Suzuki R, Muramatsu M, Kuramochi K, Sureau C, Parhami F, Watashi K. 2023. Selective inhibition of hepatitis B virus internalization by oxysterol derivatives. Biochem Biophys Res Commun 675:139–145. doi:10.1016/j.bbrc.2023.07.014 37473528

[23] Gumbiner B, Lowenkopf T, Apatira D. 1991. Identification of a 160-kDa polypeptide that binds to the tight junction protein ZO-1. Proc Natl Acad Sci USA 88:3460–3464. doi:10.1073/pnas.88.8.3460 2014265 PMC51467

[24] Jesaitis LA, Goodenough DA. 1994. Molecular characterization and tissue distribution of ZO-2, a tight junction protein homologous to ZO-1 and the Drosophila discs-large tumor suppressor protein. J Cell Biol 124:949–961. doi:10.1083/jcb.124.6.949 8132716 PMC2119984

[25] Itoh M, Morita K, Tsukita S. 1999. Characterization of ZO-2 as a MAGUK family member associated with tight as well as adherens junctions with a binding affinity to occludin and alpha catenin. J Biol Chem 274:5981–5986. doi:10.1074/jbc.274.9.5981 10026224

[26] Shin K, Fogg VC, Margolis B. 2006. Tight junctions and cell polarity. Annu Rev Cell Dev Biol 22:207–235. doi:10.1146/annurev.cellbio.22.010305.104219 16771626

[27] Niessen CM. 2007. Tight junctions/adherens junctions: basic structure and function. J Invest Dermatol 127:2525–2532. doi:10.1038/sj.jid.5700865 17934504

[28] González-Mariscal L, Gallego-Gutiérrez H, González-González L, Hernández-Guzmán C. 2019. ZO-2 is a master regulator of gene expression, cell proliferation, cytoarchitecture, and cell size. Int J Mol Sci 20:4128. doi:10.3390/ijms20174128 31450555 PMC6747478

[29] Beutel O, Maraspini R, Pombo-García K, Martin-Lemaitre C, Honigmann A. 2019. Phase separation of zonula occludens proteins drives formation of tight junctions. Cell 179:923–936. doi:10.1016/j.cell.2019.10.011 31675499

[30] Hernandez S, Chavez Munguia B, Gonzalez-Mariscal L. 2007. ZO-2 silencing in epithelial cells perturbs the gate and fence function of tight junctions and leads to an atypical monolayer architecture. Exp Cell Res 313:1533–1547. doi:10.1016/j.yexcr.2007.01.026 17374535

[31] Raya-Sandino A, Castillo-Kauil A, Domínguez-Calderón A, Alarcón L, Flores-Benitez D, Cuellar-Perez F, López-Bayghen B, Chávez-Munguía B, Vázquez-Prado J, González-Mariscal L. 2017. Zonula occludens-2 regulates Rho proteins activity and the development of epithelial cytoarchitecture and barrier function. Biochim Biophys Acta Mol Cell Res 1864:1714–1733. doi:10.1016/j.bbamcr.2017.05.016 28554775

[32] Islas S, Vega J, Ponce L, González-Mariscal L. 2002. Nuclear localization of the tight junction protein ZO-2 in epithelial cells. Exp Cell Res 274:138–148. doi:10.1006/excr.2001.5457 11855865

[33] Stieger B. 2011. The role of the sodium-taurocholate cotransporting polypeptide (NTCP) and of the bile salt export pump (BSEP) in physiology and pathophysiology of bile formation. Handb Exp Pharmacol 201:205–259. doi:10.1007/978-3-642-14541-4_5 21103971

[34] Schulze A, Mills K, Weiss TS, Urban S. 2012. Hepatocyte polarization is essential for the productive entry of the hepatitis B virus. Hepatology 55:373–383. doi:10.1002/hep.24707 21953491

[35] Appelman MD, Robin MJD, Vogels EWM, Wolzak C, Vos WG, Vos HR, Van Es RM, Burgering BMT, Van de Graaf SFJ. 2020. The lipid raft component stomatin interacts with the Na^+^ taurocholate cotransporting polypeptide (NTCP) and modulates bile salt uptake. Cells 9:986. doi:10.3390/cells9040986 32316189 PMC7226988

[36] Hu Q, Zhang F, Duan L, Wang B, Ye Y, Li P, Li D, Yang S, Zhou L, Chen W. 2020. E-cadherin plays a role in hepatitis B virus entry through affecting glycosylated sodium-taurocholate cotransporting polypeptide distribution. Front Cell Infect Microbiol 10:74. doi:10.3389/fcimb.2020.00074 32175289 PMC7056903

[37] Leckband DE, de Rooij J. 2014. Cadherin adhesion and mechanotransduction. Annu Rev Cell Dev Biol 30:291–315. doi:10.1146/annurev-cellbio-100913-013212 25062360

[38] Yap AS, Gomez GA, Parton RG. 2015. Adherens junctions revisualized: organizing cadherins as nanoassemblies. Dev Cell 35:12–20. doi:10.1016/j.devcel.2015.09.012 26460944

[39] Indra I, Choi J, Chen CS, Troyanovsky RB, Shapiro L, Honig B, Troyanovsky SM. 2018. Spatial and temporal organization of cadherin in punctate adherens junctions. Proc Natl Acad Sci USA 115:E4406–E4415. doi:10.1073/pnas.1720826115 29691319 PMC5948979

[40] Davit-Spraul A, Gonzales E, Baussan C, Jacquemin E. 2009. Progressive familial intrahepatic cholestasis. Orphanet J Rare Dis 4:1. doi:10.1186/1750-1172-4-1 19133130 PMC2647530

[41] Sambrotta M, Strautnieks S, Papouli E, Rushton P, Clark BE, Parry DA, Logan CV, Newbury LJ, Kamath BM, Ling S, Grammatikopoulos T, Wagner BE, Magee JC, Sokol RJ, Mieli-Vergani G, Smith JD, Johnson CA, McClean P, Simpson MA, Knisely AS, Bull LN, Thompson RJ, University of Washington Center for Mendelian Genomics. 2014. Mutations in TJP2 cause progressive cholestatic liver disease. Nat Genet 46:326–328. doi:10.1038/ng.2918 24614073 PMC4061468

[42] Keitel V, Burdelski M, Warskulat U, Kühlkamp T, Keppler D, Häussinger D, Kubitz R. 2005. Expression and localization of hepatobiliary transport proteins in progressive familial intrahepatic cholestasis. Hepatology 41:1160–1172. doi:10.1002/hep.20682 15841457

[43] Nishitsuji H, Ujino S, Shimizu Y, Harada K, Zhang J, Sugiyama M, Mizokami M, Shimotohno K. 2015. Novel reporter system to monitor early stages of the hepatitis B virus life cycle. Cancer Sci 106:1616–1624. doi:10.1111/cas.12799 26310603 PMC4714683

[44] Xu L, Ihara KI, Yoshimura S, Konno D, Tachibana A, Nakanishi T, Tachibana T. 2020. Generation of the rat monoclonal antibody against the extracellular domain of human CD63 by DNA immunization. Monoclon Antib Immunodiagn Immunother 39:74–76. doi:10.1089/mab.2020.0007 32311306

