# Supplementary figures and images for: ZO-2 determines cell membrane localization of receptor NTCP and supports hepatitis B virus infection
Source: mBio. 2026 Mar 23;17(4):e02704-25. doi: 10.1128/mbio.02704-25 (PMC13059785; doi:10.1128/mbio.02704-25)

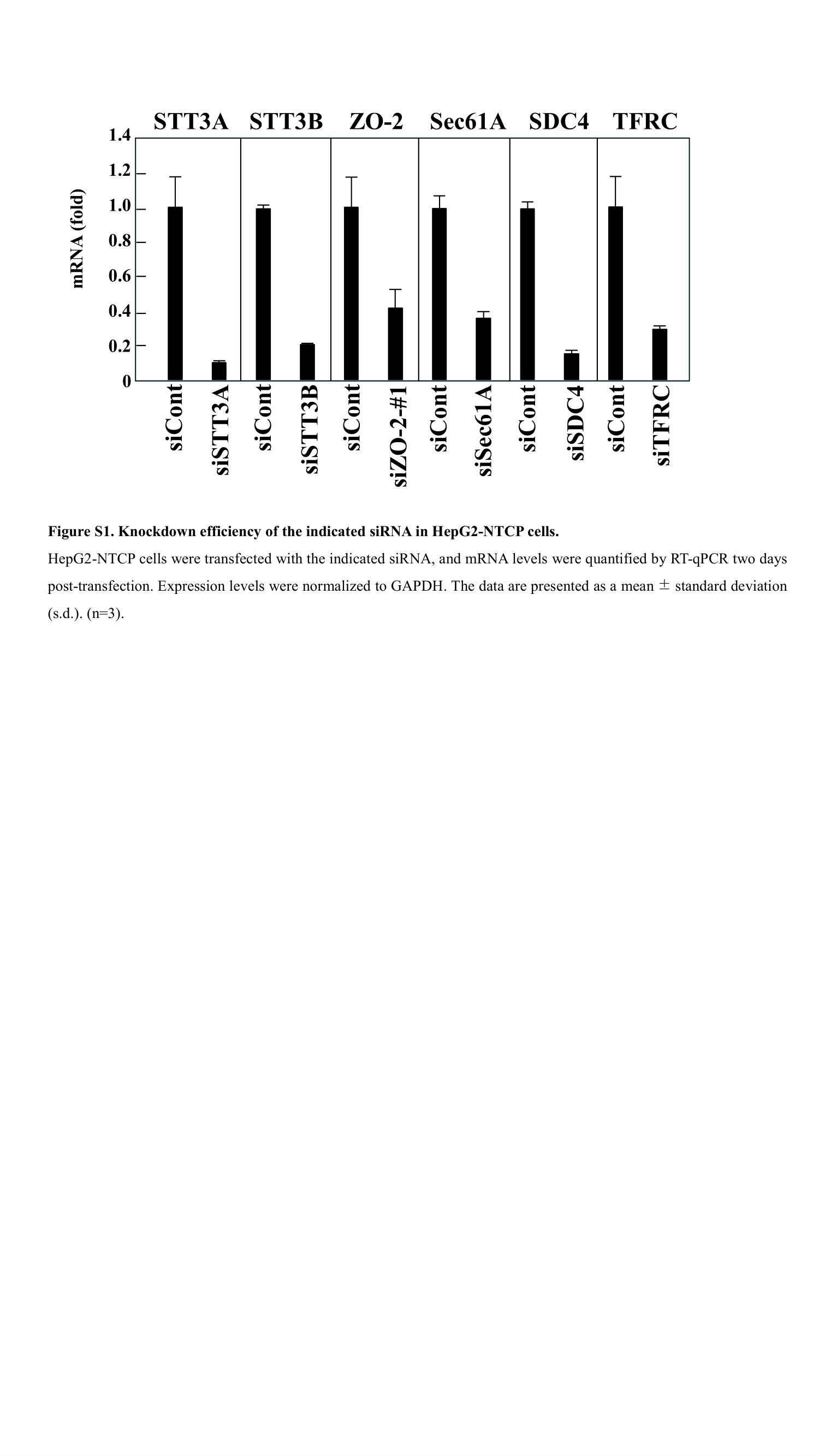

Supplement: Fig. S1 — Knockdown efficiency of the indicated siRNA in HepG2-NTCP cells. [file mbio.02704-25-s0001.tiff]

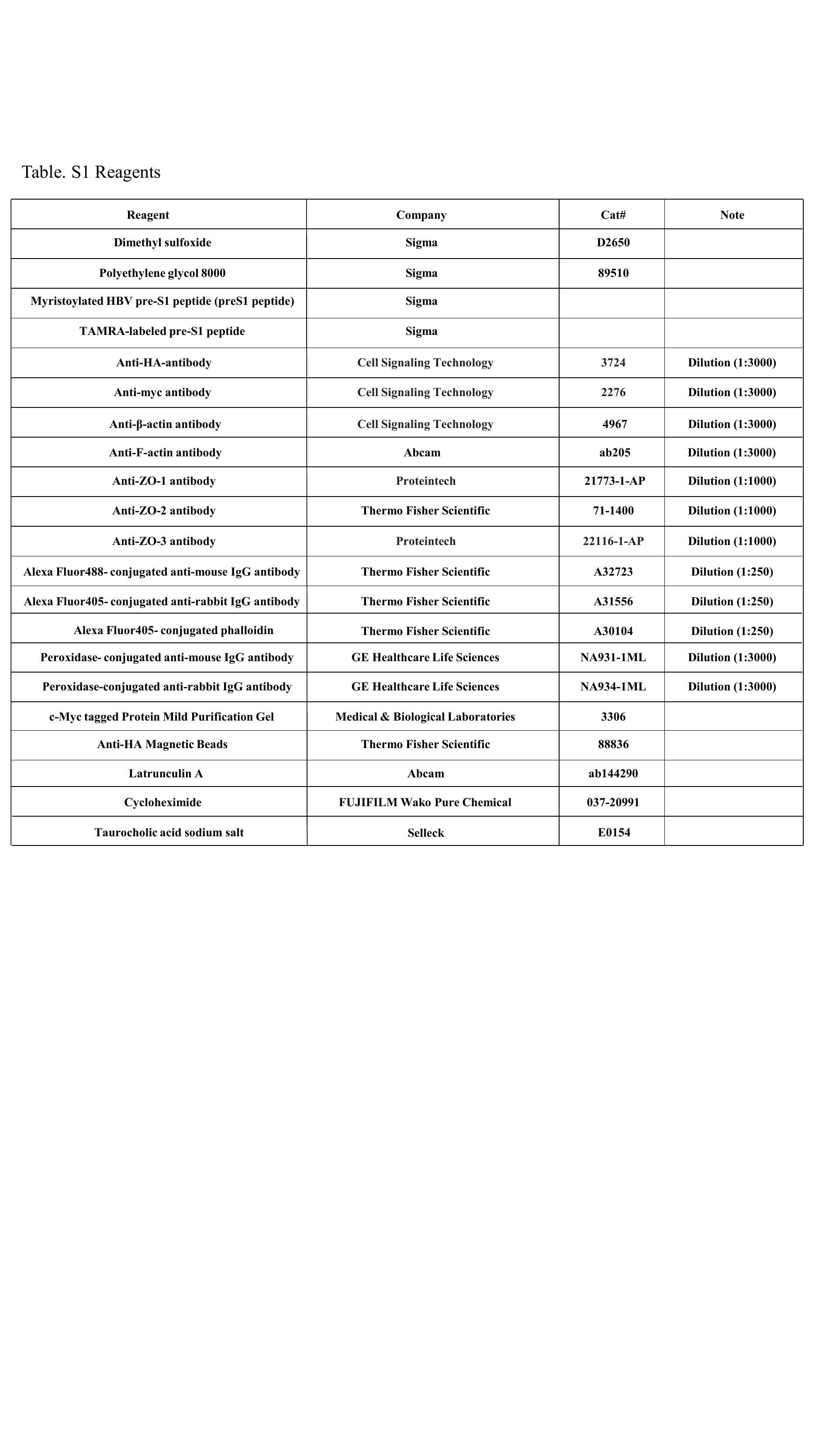

Supplement: Table S1 — Reagents. [file mbio.02704-25-s0003.tiff]
